# Kinetic Activity of Chromosomes and Expression of Recombination Genes in Achiasmatic Meiosis of *Tityus* (*Archaeotityus*) Scorpions

**DOI:** 10.3390/ijms23169179

**Published:** 2022-08-16

**Authors:** Bruno Rafael Ribeiro de Almeida, Renata Coelho Rodrigues Noronha, Adauto Lima Cardoso, Cesar Martins, Jonas Gama Martins, Rudi Emerson de Lima Procópio, Cleusa Yoshiko Nagamachi, Julio Cesar Pieczarka

**Affiliations:** 1 Laboratório de Citogenética, Centro de Estudos Avançados da Biodiversidade, Instituto de Ciências Biológicas, Universidade Federal do Pará, Avenida Perimetral da Ciência, km 01, Guamá, Belem 66075-750, PA, Brazil; 2 Instituto Federal de Educação, Ciência e Tecnologia do Pará, Campus Itaituba, R. Universitário, s/n, Maria Magdalena, Itaituba 68183-300, PA, Brazil; 3 Laboratório Genômica Integrativa, Departamento de Morfologia, Instituto de Biociências, Universidade Estadual Paulista, Distrito de Rubião Júnior, s/n, Rubião Júnior, Botucatu 18618970, SP, Brazil; 4 Pós-Graduação em Genética, Conservação e Biologia Evolutiva, Instituto Nacional de Pesquisas da Amazônia, Avenida André Araújo, 2936-Petrópolis, Manaus 69067-375, AM, Brazil; 5 Programa de Pós-Graduação em Biotecnologia e Recursos Naturais da Amazônia, Universidade do Estado do Amazonas (UEA), Avenida Carvalho Leal, 1777-Cachoeirinha, Manaus 69065-170, AM, Brazil

**Keywords:** scorpions, achiasmatic meiosis, telokinetic behavior

## Abstract

Several species of *Tityus* (Scorpiones, Buthidae) present multi-chromosomal meiotic associations and failures in the synaptic process, originated from reciprocal translocations. Holocentric chromosomes and achiasmatic meiosis in males are present in all members of this genus. In the present study, we investigated synapse dynamics, transcriptional silencing by γH2AX, and meiotic microtubule association in bivalents and a quadrivalent of the scorpion *Tityus maranhensis*. Additionally, we performed RT-PCR to verify the expression of mismatch repair enzymes involved in crossing-over formation in *Tityus silvestris* gonads. The quadrivalent association in *T. maranhensis* showed delay in the synaptic process and long asynaptic regions during pachytene. In this species, γH2AX was recorded only at the chromosome ends during early stages of prophase I; in metaphase I, bivalents and quadrivalents of *T. maranhensis* exhibited binding to microtubules along their entire length, while in metaphase II/anaphase II transition, spindle fibers interacted only with telomeric regions. Regarding *T. silvestris*, genes involved in the recombination process were transcribed in ovaries, testes and embryos, without significant difference between these tissues. The expression of these genes during *T. silvestris* achiasmatic meiosis is discussed in the present study. The absence of meiotic inactivation by γH2AX and holo/telokinetic behavior of the chromosomes are important factors for the maintenance of the quadrivalent in *T. maranhensis* and the normal continuation of the meiotic cycle in this species.

## 1. Introduction

The centromere is the region of the genome in which the kinetochore is inserted, whose function is to allows the union of the spindle microtubules to the chromosomes and promote their segregation during cell division [1]. In several lineages of arthropods, nematodes, plants, and protists, holocentric chromosomes are observed, whose kinetochore proteins are distributed along their length [2]. In mitosis, this chromosomal form is identified by the absence of primary constriction [3]. Another characteristic attributed to mitotic holocentric chromosomes is the holokinetic behavior of sister chromatids, which migrate in a parallel arrangement to each other at the cell poles during anaphase [2]. The union of microtubules along the chromosomes also helps in the conservation of fragments generated by fission, which in this way are transmitted to daughter cells [4].

Scorpions *Tityus* (Buthidae) have holocentric chromosomes, with extensive karyotype reorganization generated by reciprocal translocations and fusions/fissions [5,6,7]. Direct consequences of such alterations are observed during the meiotic events of prophase I of these arachnids, since anomalies in the pairing and synapse of homologs are recorded, for example, asynaptic regions, loop-like structures, and high frequency of heterosynapses [5,8,9,10], as well as the formation of multi-chromosomal associations during metaphase I [7,11]. Thus, the presence of trivalents, quadrivalents, and other complex forms of pairing is commonly observed in several members of the *Tityus* genus [9,12]. In these species, changes in the synaptonemal complex (gaps, non-synaptic lateral elements) were observed using ultrastructural microscopy [10]. Analysis of the behavior of the synaptonemal complex in *Tityus* and other arthropods can also be inferred by the structural maintenance of chromosome protein 3 (SMC3) immunofluorescence, a component of the cohesins axis that bind to the lateral elements during prophase I [13]. Despite this, to date, no study has investigated the occurrence of meiotic silencing of unsynapsed chromatin (MSUC) during *Tityus* prophase I. This phenomenon has been investigated in mice [14], pigs [15], birds [16], anurans [17], and insects [18], among other organisms. MSUC is important for spermatogenesis progression by signaling failures in the synaptic process and in the repair of double strand-breaks (DSBs) and consists of recruitment of proteins such as BRCA1 and ATR, which generate chromatin remodeling through γH2AX phosphorylation, inactivating genes present in asynaptic chromosomal regions [19].

The alternate segregation of elements of these meiotic associations in *Tityus* has been demonstrated through the analysis of cells in metaphase II [6,7]. In organisms bearing holocentric chromosomes, the geometry of quadrivalent associations (originated via reciprocal translocation between two nonhomologous pairs) may require modifications in the structure or behavior of the chromosomes rearranged during meiosis I to segregate correctly, similar to cruciform holocentric meiotic bivalents [20]. In plants and invertebrates, the presence of inverted meiosis [21], redistribution of CENH3 proteins [22], or restriction of kinetic activity has been reported; in the latter, only the chromosome ends join the spindle microtubules [23].

Achiasmate meiosis was previously confirmed to be associated only with heterogametic sex in plants, tardigrades, crustaceans, insects, and arachnids or in both gonads (testis and ovary) of some annelids, flatworms, and hermaphroditic mollusks [24]. Among scorpion species, cytogenetically investigated males showed achiasmatic meiosis [10,12,13]. Regarding females, the presence of chiasmata is questionable, most being considered achiasmatic, with only one case of chiasma-like structures in *Zabius* (Buthidae) [8,25,26]. The absence of chiasmata in *Tityus*, as in other genera, is considered an adaptation to the high incidence of rearrangements, since the decrease in the rate of crossing-over favors the correct segregation of homologous components of meiotic multivalents [10,13]. The molecular processes that generate achiasmatic meiosis are poorly understood and vary among the groups mentioned above. In *Drosophila*, male achiasmia may result from the differential expression of several recombination genes between the sexes [27]. In the scorpion *Tityus silvestris*, initial events of the recombination process, such as the formation of DSBs and their association with Rad51, occur during zygotene and pachytene [13]. However, no study has evaluated the activity of mismatch repair enzymes (MLH1- MutL homolog 1, MLH3- MutL homolog 3, MSH4- MutS homolog 4, MSH5- MutS homolog 5), and MUS81 (crossover junction endonuclease MUS81) in this species, which are responsible for stabilizing and resolving the Holliday junction, created from the action of Rad51 and Dmc1, giving rise to recombinant chromosomes [28].

In the present study, we evaluated through immunocytogenetics the synaptic behavior, occurrence of MSUC by γH2AX, and the distribution pattern of tubulin along bivalents and quadrivalents of the scorpion *Tityus* (*Archaeotityus*) *maranhensis*. Additionally, we investigated by RT-PCR the expression of meiotic proteins involved in the process of crossing-over formation in *Tityus* (*Archeotityus*) *silvestris*. The results obtained contributed to a better understanding of the molecular and cytological mechanisms that maintain the achiasmatic and holocentric system of Buthidae scorpions.

## 2. Results

### 2.1. Karyotype and Synaptonemal Complex Formation in T. maranhensis

**Figure 1:**
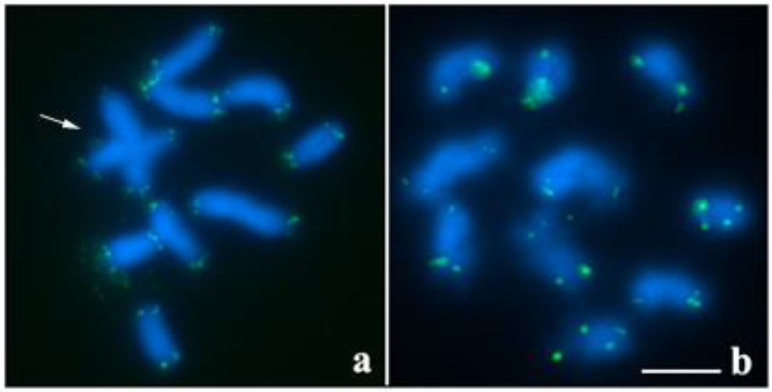
FISH with telomeric probe in meiotic chromosome of *T. maranhensis* (blue: DAPI; green: telomeres): (**a**) metaphase I (arrow indicates quadrivalent); (**b**) metaphase II. Bar: 10 µm.

The chromosomes of *T. maranhensis* did not show primary constriction, typical of holocentric condition (Figure 1). The karyotype of this species showed diploid number 2n = 20 (Figure 1a). During metaphase I, the two males of *T. maranhensis* had one quadrivalent and eight bivalents (Figure 1a), while in metaphase II, only 10 chromosomes were observed (Figure 1b). Telomeric sequences were recorded only at the chromosome ends (Figure 1a,b).

**Figure 2:**
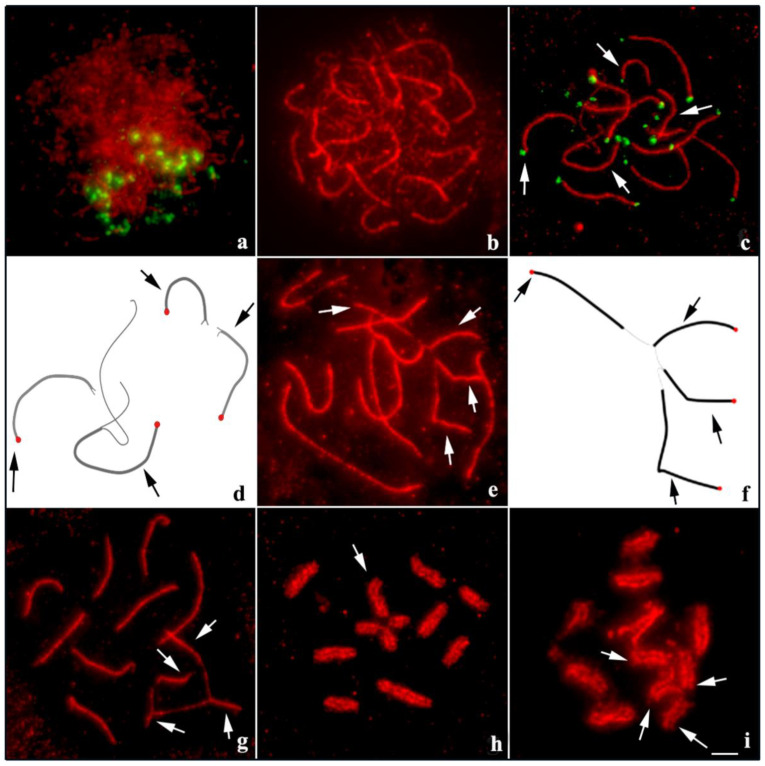
Organization of the synaptonemal complex in *T. maranhensis*. Immuno-FISH with SMC3 (red) and telomeres (green) at (**a**,**c**). (**a**) Leptotene/zygotene transition; SMC3 axes and telomeres arranged in *bouquet* configuration. (**b**) Late zygotene; synaptic and asynaptic segments of the synaptonemal complex were characterized by the presence, respectively, of thicker and thinner SMC3 filaments. (**c**) Early pachytene (arrows indicate asynaptic regions of the quadrivalent). (**d**) Schematic representation of SMC3 axes (black) and telomeres (red) pointed by arrows in (**c**). (**e**) Intermediate pachytene (arrows indicate regions of the quadrivalent in synapse process). (**f**) Schematic representation of the SMC3 axes (black) indicated by the arrows in (**e**). (**g**) Late pachytene (arrows indicate regions of the quadrivalent in synapse process). (**h**) Metaphase I (arrow indicates the quadrivalent). (**i**) Early anaphase I (arrows indicate quadrivalent components initiating segregation). Bar: 10 µm.

Meiotic analysis in *T. maranhensis* revealed that in the leptotene/zygotene transition, the terminal region of SMC3 axes (evidenced by telomeres) were polarized in the cell nucleus (Figure 2a). In late zygotene, synapse advancement was observed along the chromosomes (Figure 2b). In early pachytene, bivalents were noted to complete the synapse, while quadrivalent components still had long asynaptic regions (Figure 2c,d). This asynaptic condition of the quadrivalent in relation to bivalents was observed until the end of the pachytene stage (Figure 2e–g). During metaphase I, the SMC3 axis was visible along the bivalents and quadrivalent, suggesting that in both synaptic conditions, the synaptonemal complex remained preserved (Figure 2h). At the beginning of anaphase I, early dissociation of the quadrivalent in relation to the bivalents was also recorded (Figure 2i).

### 2.2. Distribution of γH2AX throughout Prophase I of T. maranhensis

**Figure 3:**
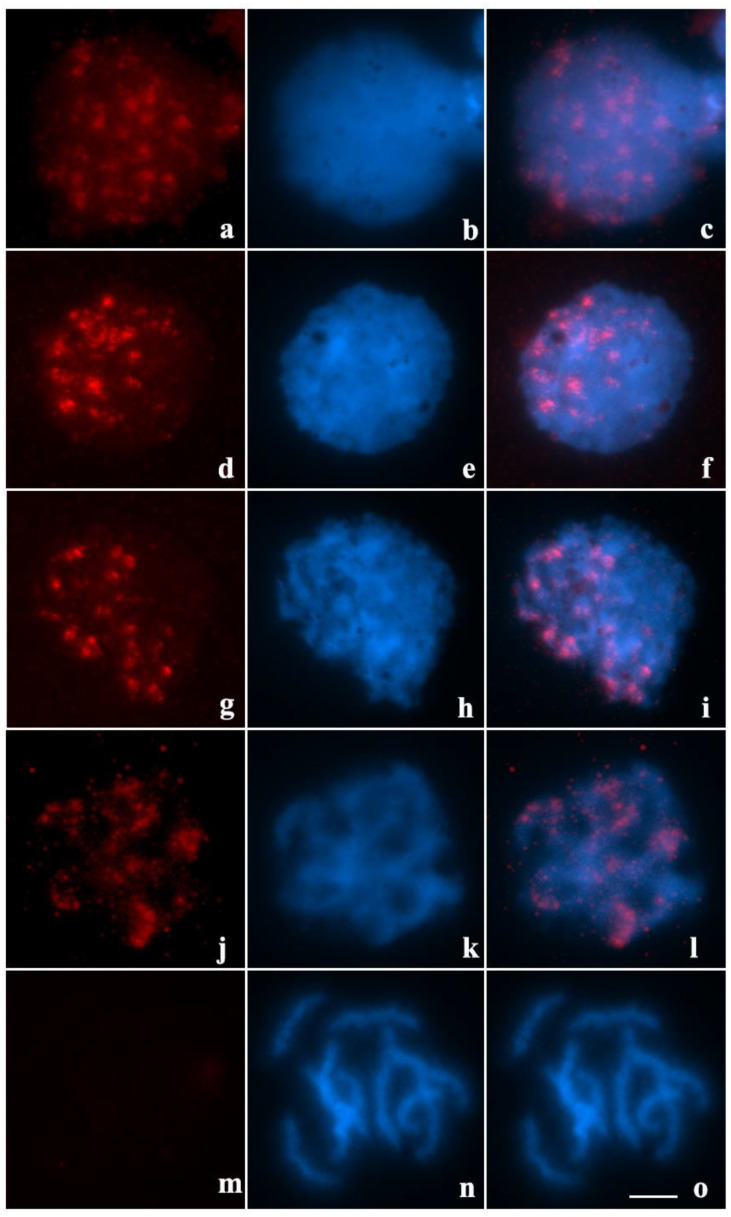
Visualization of DSBs through immunodetection of *γ*H2AX in prophase I of *T. maranhensis* (blue: DAPI; red: *γ*H2AX): (**a**–**c**) leptotene; (**d**–**f**) early zygotene; (**g**–**i**) late zygotene; (**j**–**l**) early/intermediate pachytene; (**m**–**o**) late pachytene. Bar: 10µm.

Cells in leptotene evidenced conspicuous *γ*H2AX signals throughout the nucleus (Figure 3a–c). During the leptotene/zygotene transition, *γ*H2AX were observed organized near the periphery of the cell nucleus (Figure 3d–f). At the end of the zygotene, γH2AX signals were evident on chromatin more densely stained with DAPI, indicating regions of pairing/synapse (Figure 3g–i). Cells in early stages of pachytene showed highly concentered γH2AX near the terminal regions of chromosomes (Figure 3j–l). In late pachytene and post-pachytene stages, no γH2AX signals were observed (Figure 3m–o).

### 2.3. Binding of Microtubules along the Chromosomes of T. maranhensis

**Figure 4:**
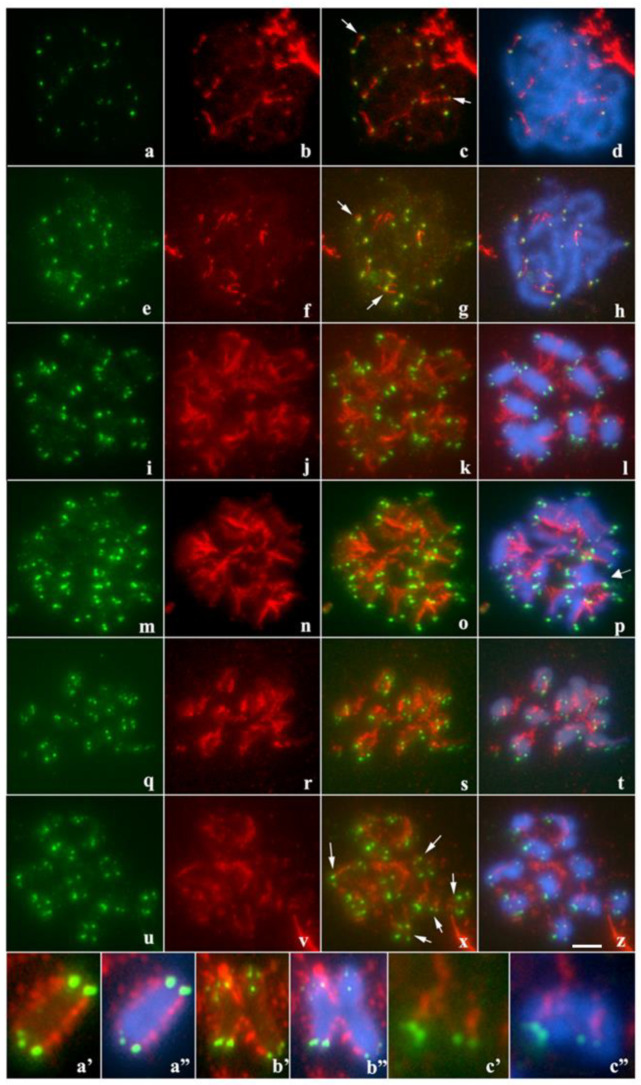
Immunodetection of tubulin along prophase I in *T. maranhensis*. In red tubulin and in green telomeres: (**a**–**d**) zygotene (arrows indicate union of telomeres and tubulin fibers); (**e**–**h**) pachytene (arrows indicate union of telomeres and tubulin fibers); (**i**–**l**) metaphase I; (**m**–**p**) anaphase I (arrow indicates quadrivalent). (**q**–**t**) metaphase II; (**u**–**z**) anaphase II (arrows indicate union of telomeres and tubulin fibers). Panel with enlarged bivalent (**a’**,**a”**) and quadrivalent (**b’**,**b”**) metaphase I; (**c’**,**c”**) sister chromatids at the beginning of anaphase II. In **a’**–**c”** tubulin (red), telomeres (green) and DAPI-stained chromosomes (blue). Bar: 10 µm.

The dynamics of microtubule binding to the kinetochore was evaluated in bivalent and quadrivalent of *T. maranhensis*. During zygotene, tubulin fibers were observed to interact exclusively with telomeric regions (Figure 4a–d). In pachytene, this pattern still occurred, although some microtubules associated with non-terminal regions were visualized (Figure 4e–h). On the other hand, in metaphase I, spindle fibers were visualized along the bivalents as well as in the four component chromosomes of the quadrivalent (Figure 4i–l,a’–b”). No differential distribution of microtubules between terminal and interstitial regions was observed at this stage (Figure 4i–l,a’–b”). This pattern was also recorded in anaphase I (Figure 4m–p). In metaphase II, our results showed that microtubules united both along the chromosomes and at the ends (Figure 4q–t). In anaphase II, the spindle fibers were visualized only interacting with telomeres (Figure 4u–z,c’,c”).

**Figure 5:**
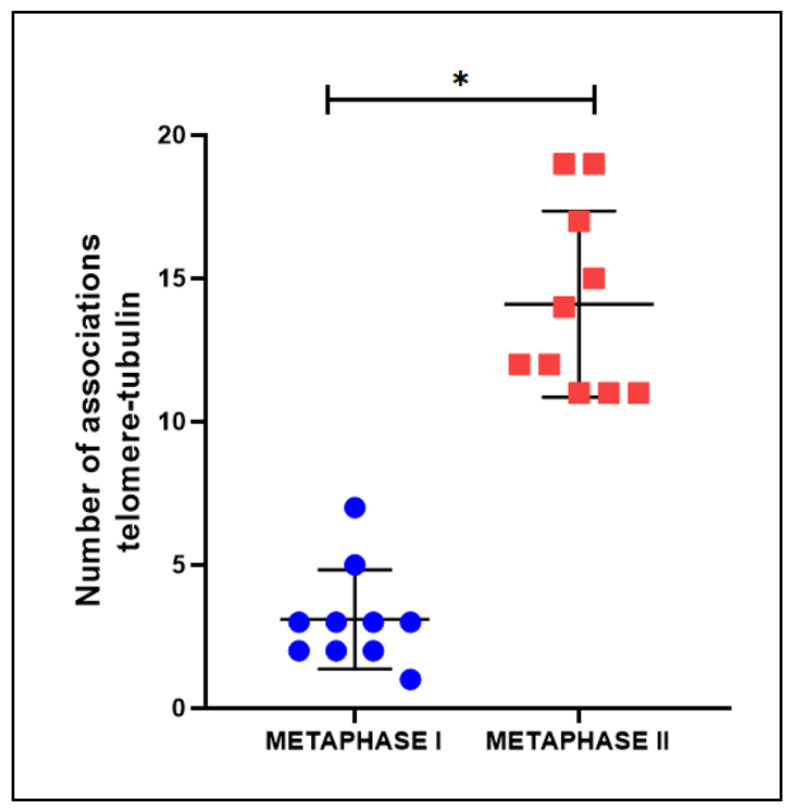
Quantification of associations of telomere–tubulin in metaphase I and metaphase II of *T. maranhensis* (n = 10 of each). (* *p* < 0.001).

To confirm the divergence regarding the distribution of tubulin along the chromosomes during meiosis of *T. maranhensis*, we quantified the number of associations between telomeres and tubulin in metaphases I and II (Figure 5). In metaphase I, an average of 3.1 ± 1729 was observed, while in metaphase II, we obtained an average of 14.10 ± 3247. When compared using the unpaired Student’s t-test, they were statistically significant (*p* < 0.001) (Figure 5).

### 2.4. Expression of Mismatch Repair Enzymes in T. silvestris

**Figure 6:**
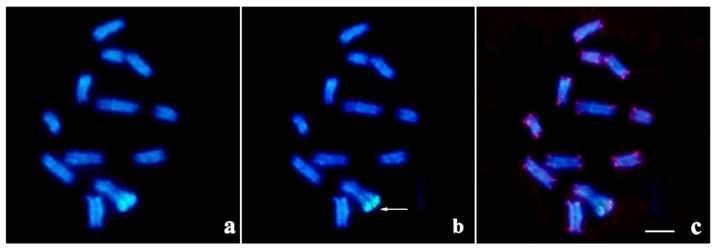
Meiotic chromosomes in cytotype A (2n = 24) of *T. silvestris* (blue: DAPI; green: 45S rDNA; red: telomeres): (**a**) C-banding; (**b**) FISH with 45S rDNA probe (arrow); (**c**) FISH with telomeric probe. Bar: 10 µm.

Specimens of *T. silvestris* from the municipalities of Manaus-AM and Belém-PA showed a karyotype consisting of 2n = 24 (Figure 6a). In meiosis I, 12 regular achiasmatic bivalents were observed in male individuals (Figure 6a). A conspicuous heterochromatic block was recorded in pair 1 (Figure 6a), co-localized with 45S rDNA (Figure 6b). Telomeric sequences were recorded only at chromosomal ends (Figure 6c).

**Figure 7:**
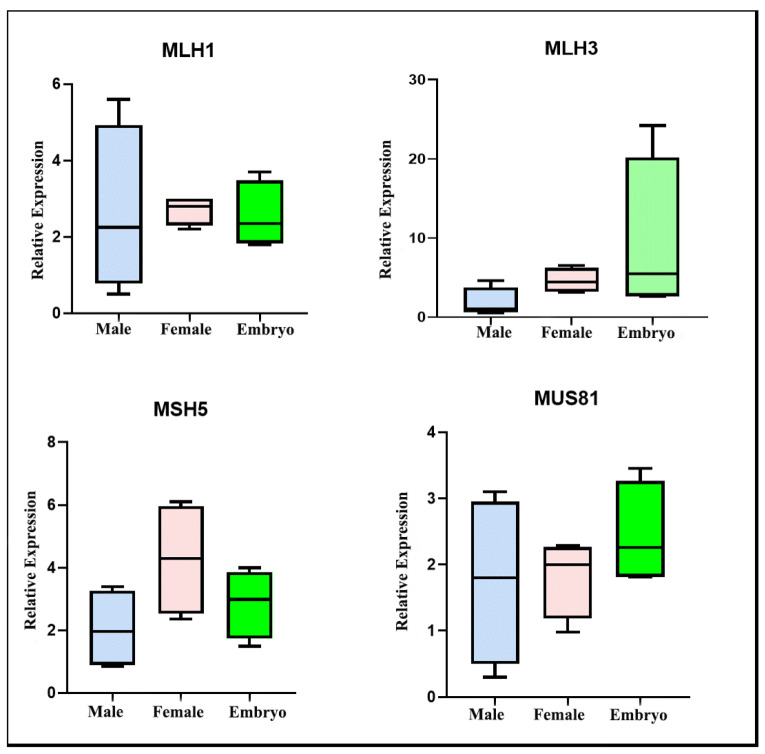
Relative expression of mismatch repair genes and MUS81 in gonads and embryos of *T. silvestris* (2n = 24). Expression values were obtained using the transcription of beta-actin gene as normalizer.

To evaluate differential expression of repair enzymes involved in crossing over formation (MLH1, MLH3, MSH4, MSH5, and Mus81) between sexes and different developmental stages, we performed RT-PCR in embryos and between both adult gonads (testis and ovary) of *T. silvestris* individuals. Our data showed expression of all genes studied in the three types of tissues analyzed, although no statistically significant difference was detected between the tissues studied (Figure 7). Nevertheless, regarding the MLH3 gene, we observed a lower expression in gonads compared to embryonic tissues, suggesting a tendency of decreased transcription of this gene throughout the development of *T. silvestris* (Figure 7).

## 3. Discussion

The diploid numbers recorded in the present study for *T. maranhensis* and *T. silvestris* are in agreement with those already described in the literature [9,13]. In several genera of Buthidae, meiotic multi-chromosomal associations, as observed in *T. maranhensis*, are frequently documented [6,12,27]. They originate through reciprocal translocations and their constitution may show geographic variation [7,9]. Analysis of the synaptonemal complex in specimens of *T. maranhensis* showed delay in the synaptic process of quadrivalent compared to bivalent. This feature has been previously reported in other species of scorpions [10,11], insects [29], mammals [30,31], and amphibians [17]. This phenomenon can be explained by mechanical stress, generated by telomere movements of chromosomes involved in multivalent, associated with regions far from the nuclear envelope [32]. Additionally, we suggest that two other events may be contributing (at distinct stages of prophase I) to the delay in quadrivalent synapse in this species: (1) resolution of interlocks and other failures in the pairing process during zygotene; (2) lack of homology between translocated segments during pachytene, as recorded previously [9].

In several animal taxa, asynaptic regions of sex chromosomes or autosomes, involved in multivalents, are transcriptionally inactivated during prophase I [17,33,34]. In mammals, this process involves the BRCA1 repair protein, which recruits the ATR kinase, responsible for phosphorylating histone H2A making it *γ*H2AX, the main epigenetic mark of meiotic chromatin silencing [35,36]. Our results showed that *γ*H2AX signals are present in terminal regions during zygotene and pachytene, but do not co-localize with asynaptic regions of the quadrivalent in *T. maranhensis*. In other arthropods, despite the occurrence of γH2AX during the early stages of prophase I, transcriptional activity of autosomes remains constant from leptotene to diakinesis, differing from the pattern observed in vertebrates [13,37,38]. Thus, we propose that this histone variant does not perform MSUC on multivalent asynaptic chromosomal segments in *T. maranhensis*. On the other hand, similar distribution pattern of this epigenetic mark was observed during meiosis in *T. silvestris* [13]. Similar to *T. silvestris*, in the present study, *γ*H2AX was associated with chromosomal regions that initiate the synapse process and were not observed at late pachytene stages, where the quadrivalent is not yet fully synapsed (Figure 2e–g); for this reason, we suggest that γH2AX signals in *T. maranhensis* are related to sites of SPO11-induced DSBs, which correspond to early events in the meiotic recombination process, with an important role for homolog pairing.

In *T. maranhensis*, we observed distinct forms of attachment of microtubules to meiotic chromosomes. In zygotene and pachytene, the association of spindle fibers to telomeres denotes the action of microtubules in the mobility of these genomic regions during organization/disorganization of the telomeric bouquet and homologous pairing [39]. In metaphase I, the pattern of tubulin binding to *T. maranhensis* bivalents corresponds to that expected for holokinetic chromosomes [21,40]. Regarding the quadrivalent observed in this species, we initially hypothesized that this association might require meiotic adaptations, similar to those observed for holocentric bivalents with cruciform configuration [23,41]. However, we visualized adhered tubulin fibers along the entire length of the chromosomes involved in the meiotic quadrivalent of *T. maranhensis*. On the other hand, *T. maranhensis* (present study) showed chromosomes with telokinetic behavior in metaphase II/anaphase II. Restriction of kinetic activity is observed in both metaphases I and II in nematodes and insects of the orders Hemiptera and Odonata [42]. The molecular mechanisms responsible for this phenomenon are poorly understood. In most organisms with restricted kinetochore activity, CENH3 and kinetochore are noted to be absent during meiosis, and thus microtubules are inserted directly into chromatin, interacting especially with terminal heterochromatic regions [43,44,45]. These studies suggest that the kinetochore may be dispensable for meiotic segregation in holocentric organisms with telokinetic activity. In *Tityus bahiensis*, the kinetochore is present in both metaphase I and II, but does not completely cover the chromosomes [46]. The functional significance of telokinetic activity in meiosis II in *T. maranhensis* is not yet understood, but it may be related to an intrinsic process of degradation of the cohesin complex present between the sister chromatids of the chromosomes of this species, which allow their correct arrangement in the metaphase plate and segregation to daughter cells, similar to that described previously for *C. elegans* [47] and *Triatoma infestans* [23].

Our results revealed transcription of mismatch repair enzymes in gonads and embryos of *T. silvestris*; despite this, we did not detect a significant difference in the expression levels of these genes between sexes (see Figure 7). This result differs from the pattern observed in *Drosophila*, which shows differential expression of meiotic genes involved in the recombination process between males (achiasmatic) and females (chiasmatic) [27]. This indicates that achiasmia in *T. silvestris* and *Drosophila* may originate and be maintained by distinct processes. During meiosis I of various eukaryotes, MLH1, MLH3, MSH4, and MSH5 are components of recombination nodules, and they perform the processing of DSBs by homologous recombination, generating chiasma in bivalents during prophase I [28]. Recombination nodules are absent in *Tityus bahiensis* and *Tityus fasciolatus*, corroborating the achiasmia in *Tityus* males [10]. Similar findings have been reported in other Scorpiones [48] and achiasmatic females of Lepidoptera [49]. Thus, considering the absence of chiasma and recombination nodule during meiosis in *Tityus* males, we conclude that mismatch repair genes are expressed in *T. silvestris* testis, but their functions related to the recombination process may be inhibited in gonads of this scorpion. The expression of Mus81 was detected in *T. silvestris*; this resolvase is not a component of recombination nodules and may promote crossing-over formation by cleaving the Holliday junction in a manner distinct from other repair enzymes [50]. Thus, its mechanism of action in achiasmatic scorpions needs to be investigated. Additionally, part of the expression of these genes observed in testis of *T. silvestris* may be related to DNA repair in spermatogonia and spermatozoa [51].

Our results confirmed the karyotype characteristics described previously for *T. maranhensis* and *T. silvestris*, with the presence of a quadrivalent meiotic association in the former, resulting from reciprocal translocation in heterozygosity. Our data allow us to conclude that (1) there is a delay in the synaptic process of quadrivalent during pachytene and post-pachytene; (2) γH2AX does not act in the inactivation of non-synapsed chromatin of multivalents of *T. maranhensis*; (3) the chromosomes of *T. maranhensis* exhibit holo- and telokinetic behavior, respectively, in metaphases I and II; (4) expression of mismatch repair genes is observed in both sexes in *T. silvestris*, despite the absence of chiasmata. Collectively, our data highlight that *Tityus* may be a model taxon for studies of achiasmatic meiosis in holocentric organisms.

## 4. Materials and Methods

### 4.1. Karyotype Analysis

Two species of *Tityus* were considered in this study. Information regarding the species, number, and sex of individuals and collection localities of the sample used in the present are described in Table 1. Taxonomic identification was performed according to the specialized literature [52]. Specimens were deposited in the collection of the Laboratory of Medical Entomology and Venomous Arthropods (LEMAP/UFPA). Gonads and embryos were hypotonized in 0.075M KCl and subsequently fixed in methanol:acetic acid solution (3:1). Cell suspension generated from digestion of gonads and embryos in 60% acetic acid was spread on slides at 45 °C. Chromosomes were stained with 5% Giemsa.

### 4.2. Probes

Probe of arthropod telomeric repeats (TTAGG) was obtained by polymerase chain reaction (PCR) using described primers [53]. The PCR reaction consisted of 17.25 µL of sterile water, 2.5 µL of 10× Taq polymerase buffer, 2 µL of DNTP mix (2 mM), 1 µL of MgCl2 (50 mM), 1 µL of forward primer (10 mM), 1 µL of reverse primer (10 mM), 0.25 µL of 1U Taq polymerase. The thermal configurations of the PCR were 1 cycle of 94 °C (5 min); 35 cycles of 94 °C (1 min), 55 °C (1 min) and 72 °C (1 min); 1 cycle of 72 °C (10 min); 1 cycle of 4 °C (hold). To obtain the 45S rDNA probe, the plasmid pTa71 [54] was used. Probes were labeled by nick translation with digoxigenin-14-dUTP (Roche, Mannheim, Germany) or biotin-11-dATP (Invitrogen, San Diego, CA, USA).

### 4.3. Immunodetection of Meiotic Proteins

Obtaining synaptonemal complexes was performed according to the literature [13]. Gonads were hypotonized with 0.075 M KCl and macerated in 200 μL of 100 mM sucrose using needles until they formed a suspension of cells, which was spread on slides previously coated with 2% paraformaldehyde (pH 8.2). Slides were incubated for 2 h in a humid chamber, then washed in Photo-Flo (pH 8.2) and stored at −80 °C.

The primary antibodies used and their respective dilutions in PBS were rabbit anti-SMC3 (Abcam, ab9263) in 1:200; mouse anti-α-tubulin (Santa Cruz, sc-23948) in 1:50; rabbit anti-γH2AX (Abcam, ab2893) in 1:50. For immunodetection of meiotic proteins, blocking with 5% BSA (containing 0.01% Triton-20, and PBS1x) was performed for 30 min. Then, the slides were incubated with primary antibodies for 2 h (37 °C). After washing in PBS1X, they were incubated with appropriate secondary antibodies, diluted 1:100, at 37 °C for 2 h. Finally, chromosomes were counterstained with DAPI, contained in VECTASHIELD antifading (Vector). Images were captured using a DS-Qi1Mc camera (Nikon, Tokyo, Japan) attached to a Nikon H550S epifluorescence microscope (Nis-Elements AR software (Nikon, Tokyo, Japan); all images were photographed using 100× magnification objective lenses.

### 4.4. Immuno-FISH

Immuno-FISH with telomeric probe was performed sequentially to the immunodetection of meiotic proteins, according to the protocol described [55]. Denaturation of chromosomal DNA and probes occurred at 70 °C and 100 °C, respectively. Slides were kept at 37 °C, overnight, for hybridization. Subsequently, slides were washed with 2xSSC and 4xSSC-Tween to remove nonspecific hybridizations. Probes were detected with anti-digoxigenin-FITC. Chromosomes were counterstained with 4-6-diamidino-2-phenylindol (DAPI) in VECTASHIELD antifading solution (Vector). Images were captured using an AxioCam MRm CCD camera (Nikon, Tokyo, Japan) attached to a Nikon H550S epifluorescence microscope (Nis-Elements AR program (Nikon, Tokyo, Japan)) and the Nis-Elements software; all images were photographed using 100× magnification objective lenses.

### 4.5. RT-PCR

RT-PCR analysis was performed to investigate the expression of MLH1, MLH3, MSH5, and MUS81 enzymes. Total RNA was isolated from embryos (n = 4), testes (n = 4), and ovaries (n = 4) of *T. silvestris* using Trizol reagent according to the manufacturer’s guidelines. After quantification and quality analysis of the RNA samples, they were treated with DNAse I. Reverse transcription was performed using the High Capacity cDNA Reverse Transcription kit from Applied Biosystems. Quantitative PCR was performed using the GoTaq qPCR Master Mix kit (Promega), using the primer sets as illustrated in Table 2. The reactions were composed of 7.5 µL of SYBR Green Mix, 0.6 µL of forward and reverse primers (400 nM), 5.8 µL of sterile water, and 0.5 µL of cDNA. The thermal conditions were 1 cycle of 95 °C for 10 min, and 40 cycles of 95 °C for 15 s and 60 °C for 1 min. Primers from the β-actin gene [56] were used endogenously in order to normalize the relative expression of the others. Measurement of expression levels was performed on the Step One Plus system (Life). Data were normalized using the Q-Gene program [57].

## Figures and Tables

**Table 1:** General data about the specimens sampled in this study.

Species	Number of Individuals	Collection Location
*Tityus silvestris* Pocock, 1897	1 male, 1 female, and 4 embryos	Manaus, Amazonas, Brazil (3°06′10″ S/59°58′42″ O)
	3 males and 3 females	Belém, Pará, Brazil (1°28′06″ S/48°26′24″ O)
*Tityus maranhensis* Lourenço de Jesus Junior e Limeira-de-Oliveira, 2006	1 male	Marapanim, Pará, Brazil (0°56′12″ S/47°38′39″ O)
	1 male and 1 female	Curuçá, Pará, Brazil (0°46′40″ S/47°49′40″ O)

**Table 2:** Primer set used for quantitative PCR.

Gene	Primers	Tm (°C)	Amplicon Size
MLH1	F:5′-GATAGCGAGGAGAGTGGATGG-3′	59.5	88 bp
	R: 5′-CCGCCTTTGTCTGTAGGAAATC-3′	59.3	
MHL3	F:5′-CCTCTGCCTTCCGAAGTTGTT-3′	60.5	118 bp
	R: 5′-CGCAAACATTCGTAGCAAAAGC-3′	59.9	
MSH5	F:5′-GCGGGACCTAACGAACATTC-3′	59	117 bp
	R: 5′-GATGTCAACGGGCAACTCG-3′	59.2	
MUS81	F:5′-CGTACCTGGATCGGGAAGC-3′	59.9	161 bp
	R:5′-AAGCAGTGTAATGGCTACCAGG-3′	60.4	
β-Actin	F:5′-TGCGGTGGACAATGGAAGG-3′	62 °C	109 bp
	R:5′-GTCTGGATTGGTGGCTCTATCT-3′	60 °C	

## Data Availability

All relevant data are found in the manuscript.
